# The *Lancet*

**Published:** 1861-09

**Authors:** 


The Lancet for September is at hand, with the first of a course of lectures on Pain, *and the Therapeutic Influence of Mechanical and Physiological* Rest *in Accidents and Surgical Diseases*, by John Hilton, Esq., F. R. S., before the Royal College of Surgeons. The usual variety of interesting papers, etc., fills the number, making the *Lancet* one of our most valued exchanges.

